# The Effect of Abnormal Regional Homogeneity and Spontaneous Low-Frequency Brain Activity on Lower Cognitive Ability: A Cross-Sectional Study on Postoperative Children With Tetralogy of Fallot

**DOI:** 10.3389/fnins.2021.685372

**Published:** 2022-02-07

**Authors:** Siyu Ma, Yuanli Hu, Yuting Liu, Yiwei Pu, Pengcheng Zuo, Qinghui Hu, Zhaocong Yang, Feng Chen, Zongyun Xie, Yueshuang Cun, Xiaoxu Liu, Ming Yang, Xuming Mo

**Affiliations:** ^1^Department of Cardiothoracic Surgery, Children’s Hospital of Nanjing Medical University, Nanjing, China; ^2^Department of Radiology, Children’s Hospital of Nanjing Medical University, Nanjing, China

**Keywords:** Tetralogy of Fallot, ALFF, ReHo, cognition, VIQ, brain injury

## Abstract

Despite intracardiac malformation correction, children with Tetralogy of Fallot (TOF) may still suffer from brain injury. This cross-sectional study was primarily designed to determine the relationship between blood oxygenation level-dependent (BOLD) signal changes after surgery and cognition in school-aged children with TOF. To evaluate the differences between TOF children (*n* = 9) and healthy children (*n* = 9), resting-state functional magnetic resonance imaging (rs-fMRI) and the Wechsler Intelligence Scale for Children–Chinese revised edition (WISC-CR) were conducted in this study. The results showed that TOF children had a lower full-scale intelligence quotient (FSIQ, 95.444 ± 5.354, *p* = 0.022) and verbal intelligence quotient (VIQ, 92.444 ± 4.708, *p* = 0.003) than healthy children (FSIQ = 118.500 ± 4.330;VIQ = 124.250 ± 4.404), and that significant differences in regional homogeneity (ReHo) and amplitude of low-frequency fluctuation (ALFF) existed between the two groups. Besides, VIQ had significantly positive correlations with the decreased ALFF value of the middle inferior occipital gyrus (MIOG, beta = 0.908, *p* = 0.012) after fully adjusting for all covariates. In addition, elevated ReHo values of the left and right precuneus were positively related to ALFF in the MIOG. This study revealed that brain injury substantially influences neural activity and cognition in postoperative TOF children, providing direct evidence of an association between BOLD signal changes and the VIQ and prompting further attention to language development in TOF children.

## Introduction

Tetralogy of Fallot (TOF) is one of the most common cyanotic congenital cardiac malformations (Diaz-Frias and Guillaume, 2020), accounting for approximately 5% of all congenital heart diseases (CHDs) (Apitz et al., 2009). TOF is characterized as a ventricular septal defect (VSD), aortic overriding, right ventricular outflow tract obstruction, and right ventricular hypertrophy (Warnes et al., 2008), and patients with TOF exhibit a decrease in systemic vascular resistance and an increase in pulmonary resistance, which leads to a right-to-left shunt in combination with a VSD and an obvious decrease in saturation (Ho et al., 2018). Less than 1% of patients survive to 40 years old naturally (Ai et al., 2018), but nearly 90% of patients with early diagnosis and surgical treatment will go on to survive (Hickey et al., 2009); however, adverse outcomes can still occur many years after cardiac surgery, including heart failure, arrhythmia, and right ventricular outflow tract reobstruction (Apitz et al., 2009). Notably, brain injury is still a non-negligible issue that persistently negatively influences survivors (Gaynor et al., 2015).

Brain injury among repaired TOF cases has recently been reported, for which children with multiple cerebral domain injuries to areas such as the cortex, basal ganglia, thalamus, and cerebral white matter account for nearly a fifth of cases (Hovels-Gurich et al., 2006; Leonetti et al., 2019). Moreover, many studies have shown that children with cerebral injuries usually have neurodevelopmental disabilities, which may manifest as cognitive impairment, oral dyskinesia, language expression abnormalities, motor delays, or attention deficit/hyperactivity disorder (ADHD) (Marino et al., 2012; Mussatto et al., 2014; Wernovsky and Licht, 2016). Although it is thought that human neurodevelopment is almost complete at the end of the second or third trimester of pregnancy, recent studies have shown that cortical neurogenesis is still active after birth (Morton et al., 2017; Ortinau et al., 2018), and that children with CHDs have delayed brain development (Clouchoux et al., 2013). Thus, early detection and intervention could afford greater neurodevelopmental benefits for children with CHDs. Fortunately, functional magnetic resonance imaging (fMRI) has been applied to identify neurodevelopmental disabilities more accurately (Ma et al., 2020).

Two blood oxygenation level-dependent (BLOD) fMRI signals, regional homogeneity (ReHo) and the amplitude of low-frequency fluctuations (ALFFs) (Zang et al., 2004, 2007), have been widely used to investigate the pathophysiology, diagnosis, and treatment effectiveness of cognitive disorders (Ni et al., 2016; Bak et al., 2018; Gur et al., 2021) and neuropsychiatric disorders, such as depression (Liu et al., 2012), schizophrenia (Guo et al., 2014; Xu et al., 2015), epilepsy (Maneshi et al., 2014), Alzheimer’s disease (Liu et al., 2014; Lyu et al., 2021), and Parkinson’s disease (Helmich et al., 2010; Li et al., 2018; Yue et al., 2020). ReHo reflects regional functional connectivity or synchronization and indicates the regional integration of information processing (Zuo et al., 2013; Jiang et al., 2015). ALFF is a method of monitoring low-frequency brain spontaneous activity and blood oxygen levels, which indicates the spontaneous activity of brain regions (Zang et al., 2007).

However, there was no research on ReHo and ALFF changes in TOF or CHD and the influence of ReHo and ALFF changes on the cognitive abilities of children following TOF repair surgery remains to be elucidated. Our study explored ReHo and ALFF fMRI changes, evaluated the cognitive abilities of TOF children, and further identified the relationship between them. Interestingly, the results revealed that decreased ALFF values in the left middle inferior occipital gyrus are correlated with a lower verbal intelligence quotient (VIQ).

## Materials and Methods

### Subjects

From November 2015 to June 2016, 9 school-aged children with TOF after repair surgery were validated as participants, and 9 healthy children (HC), identified as having no cardiovascular or nervous system diseases and matched with the TOF children by age, sex, and education, were enrolled as the control group. All TOF children underwent correction surgery at Children’s Hospital of Nanjing Medical University and were identified to be free of central nervous system diseases or hereditary syndromes, such as craniocerebral trauma, cerebral tumors, or Down syndrome. Informed consent was obtained from all the participants’ legal guardians. All children were right handed and had no known contraindications to MRI, including claustrophobia and implanted pacemakers. Finally, all 18 children underwent resting-state fMRI (rs-fMRI) examination. Additionally, all data met the standards for further analysis: All participants completed all testing tasks of Wechsler Intelligence Scale. The results need not to be calculated by alternative subtests. All children were requested to keep their eyes closed and avoid sleeping, thinking about anything, or any head motion as far as possible (less than 1 mm of translation or 1° of rotation) during the MRI scanning.

### Cognitive Ability Evaluation

The Wechsler Intelligence Scale for Children–Chinese revised edition (WISC-CR) was used to evaluate the cognitive abilities of the subjects. The WISC is an authoritative intelligence scale to assess the cognitive ability of children (Marino et al., 2012). Based on the Chinese population, the WISC-CR was adaptive for Chinese children aged 6–16, and the test contains 12 domains, including analogies, common sense, arithmetic, comprehension, vocabulary, digit span, picture arrangement, missing picture completion, block design, decoding, object collocation, and mazes (of these, digit span and mazes are optional). According to the operating manual and adjusted for the subject’s age, the subjects’ scores were calculated. The VIQ depended on the first six items, and the performance intelligence quotient (PIQ) depended on the last six items. Finally, the full-scale intelligence quotient (FSIQ) was measured.

### Functional Magnetic Resonance Imaging Data Acquisition and Preprocessing

Functional MRI scans were performed on all subjects using a 1.5T MRI scanner (Siemens MAGNETOM Trio, Erlangen, Germany) at the Radiology Department of our hospital. Earplugs and foam were used to decrease scanning noise and head motion, respectively. All subjects were not anesthetized, and were required to keep their eyes closed and avoid sleeping, thinking about anything, or any head motion as far as possible during the scanning. In total, 176 high spatial resolution T1-weighted structural images were acquired using a magnetic prepared gradient echo (GE) sequence [repetition time (TR) = 1,940 ms, echo time (TE) = 3.08 ms, field of view (FOV) = 250 mm × 250 mm, matrix = 256 × 256, slice thickness = 1 mm; flip angle = 15°], and 180 functional images by using an echo-planar imaging sequence sensitive to BOLD contrast (TR = 2,000 ms, TE = 25 ms, FOV = 240 mm × 240 mm, matrix = 64 × 64, slice thickness = 4 mm, flip angle = 90°). Twenty-two fluid attenuated inversion recovery images were used to screen for structural brain lesions by two radiology chief physicians (TR = 8,000 ms, TE = 92 ms, FOV = 220 mm × 220 mm, matrix = 512 × 464, slice thickness = 5 mm).

Preprocessing was carried out by applying DPARSFA using SPM8.^1^

We underwent preprocessing as follows:

(1) We have collected 180 points by BOLD in this study and removed the first 10 time points to reduce the influence of MRI magnetic field instability and noise during the initial scan.
(2) Slice-timing correction: we carried out slice-timing correction for the remaining time points make different layers within a TR equivalent to the same time acquisition.
(3) Head movements correction: This included translation and rotation in 3D space. Considering the long scan time and the influence of magnetic resonance noise, the purpose of head movement correction was to eliminate the tiny head movement caused by respiration, heartbeat, and other physiological factors. Subjects would be removed from this study when their head movement of x, y, or z axis were more than 1 mm of translation or 1° of rotation.
(4) Spatial registration: All MRI images were standardized to the same reference space (standard anatomical template for the head, Montreal Neurological Institute, Canada) because of the differences in brain morphology among different subjects.
(5) Linear detrending: The standardized data were then processed to remove linear trends to remove physiological linear drift, noise caused by head movement, and instability of the machine.
(6) Low-frequency filtering. A frequency band of 0.01–0.08 Hz was considered valuable physiological signals and used to filter out low-frequency drift.
(7) Spatial smoothing. A Gaussian kernel function with a full width at half maximum (FWHM) of 4 × 4 × 4 mm^3^ was used to perform spatial smoothing of fMRI images.

### Regional Homogeneity and Amplitude of Low-Frequency Fluctuation Analysis

The preprocessed fMRI data were used for the ReHo analysis of all subjects. Kendall’s coefficient concordance (KCC) was used as an indicator to measure the similarity between the time series of a voxel and those of its adjacent neighbors in the ReHo analysis. We considered 27 individual voxels as a whole and acquired ReHo maps for each participant by calculating the KCC value between each individual voxel and the other 26 adjacent voxels. ReHo images were smoothed with an isotropic Gaussian kernel of 4 mm full-width half-maximum (FWHM) to reduce space noise. The brain was divided into different regions of interest (ROI), and the mean ALFF value of each ROI was calculated with the Resting-State fMRI Data Analysis Toolkit (REST version 1.8)^2^ (Song et al., 2011). ReHo and ALFF differences between the children with TOF and the HC were analyzed with a two-sample *t*-test in REST software. AlphaSim was calibrated with *P* < 0.01 and a cluster size = 23 voxels in the ReHo analysis. AlphaSim was calibrated with *P* < 0.05 and a voxel size > 65 in the ALFF analysis.

### Statistics

SPSS 20.0 (IBM Corp., Armonk, NY, United States) was used to perform the statistical analyses in the study. We present continuous variable data as the mean ± SE in Table 1. Differences between the children with TOF and the HC were calculated by two-sample *t*-tests. Correlations between the ALFF value, ReHo value, intelligence quotient (IQ), and related covariates were investigated by using single and multiple linear regression analyses. Statistical significance was considered when *P* < 0.05.

**TABLE 1:** Characteristics of TOF and healthy children.

Variables	TOF	HC	*p*-value
		
	(*n* = 9)	(*n* = 9)	
Age (month)	121.547 ±7.811	117.560 ±3.687	0.651
Sex (male%)	66.667	55.556	0.653
Education (month)	29.427 ±4.931	28.227 ±4.412	0.858
Household income (Yuan per year)	82000.000 ±9327.379	84666.667 ± 6960.204	0.822
Age of surgery (month)	27.309 ± 7.880	NA	
Postoperative time (month)	86.674 ± 11.232	NA	
Hospital stays (day)	17.250 ± 1.943	NA	
Preoperative SpO_2_ (%)	74.286 ± 5.830	NA	
Preoperative SBP (mmHg)	100.143 ± 3.622	NA	
Preoperative DBP (mmHg)	60.000 ± 2.104	NA	
Preoperative Ph	7.339 ± 0.012	NA	
CPB time (min)	62.750 ± 2.589	NA	
AO time (min)	38.063 ± 1.848	NA	
VIQ	92.444 ± 4.708	124.250 ± 4.404	**0.003**
PIQ	97.778 ± 6.302	108.000 ± 5.492	0.342
FSIQ	95.444 ± 5.354	118.500 ± 4.330	**0.022**

*Mean ± SE.*

*TOF, Tetralogy of Fallot; HC, healthy children; SpO_2_, saturation of pulse oxygen; SBP, systolic blood pressure; DBP, diastolic blood pressure; pH, potential of hydrogen; CPB, cardiopulmonary bypass; AO, aortic occlusion; VIQ, verbal intelligence quotient; PIQ, performance intelligence quotient; FSIQ, full scale intelligence quotient; NA, not available.*

*Bold values represent that the results have statistical significance.*

## Results

The demographic characteristics of the TOF and HC groups are shown in Table 1. The summarized hospital information of the TOF children is also shown. No significant differences were observed for age, sex, education, or household income. Additionally, children with TOF had lower VIQ (92.444 ± 4.708, *P* = 0.003) and FSIQ (95.444 ± 5.354, *P* = 0.022) scores than the HC.

**FIGURE 1:**
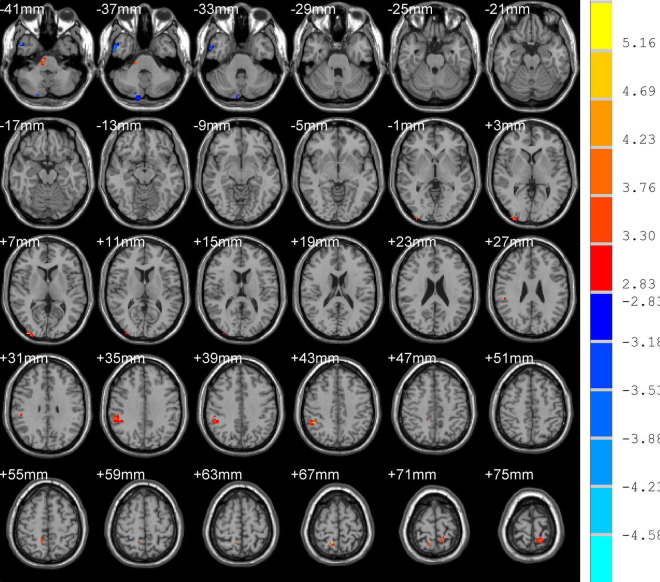
Compared with HC, TOF children showed increased ReHo values in the right brainstem, right middle occipital gyrus, right inferior parietal gyrus, right precuneus, and left precuneus, and decreased ReHo values in the right posterior lobe of the cerebellum and right inferior temporal gyrus. AlphaSim correction with *P* < 0.01; cluster size > 23.

**FIGURE 2:**
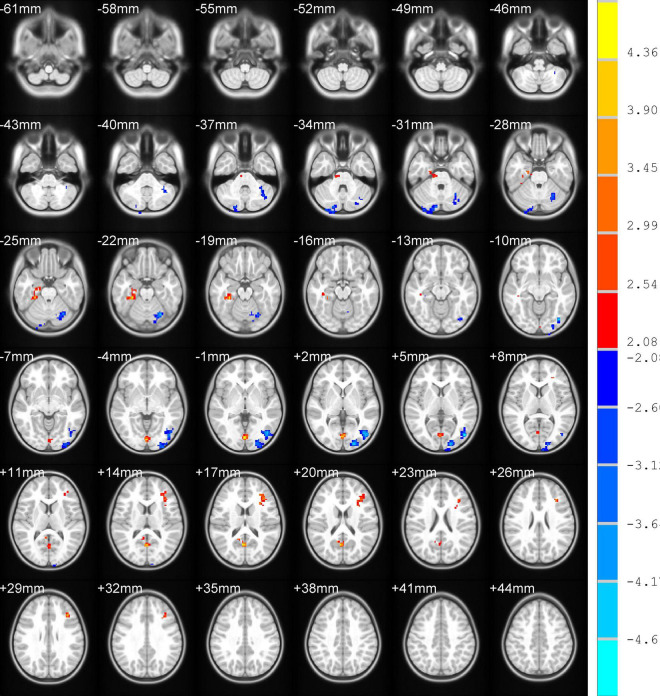
Compared with HC, TOF children had higher ALFF values in the left medial prefrontal cortex, left cingulum, and right parahippocampal gyrus, but lower ALFF values in the bilateral cerebellum, left middle inferior occipital gyrus, and left inferior occipital gyrus. AlphaSim correction with *P* < 0.05; cluster size > 65.

Differences in ReHo and ALFF are separately shown in Tables 2, 3, respectively. Compared with the HC group, ReHo values were increased in the right brainstem, right middle occipital gyrus, right inferior parietal gyrus, right precuneus, and left precuneus of the TOF group, but reduced in the right posterior lobe of the cerebellum and right inferior temporal gyrus (Table 2 and Figure 1). In addition, TOF children had higher ALFF values of the left medial prefrontal cortex, left cingulum and right parahippocampal gyrus, and lower ALFF values of the left cerebellum, left middle inferior occipital gyrus (MIOG.L), left inferior occipital gyrus, and right cerebellum (Table 3 and Figure 2).

**TABLE 2:** Cerebral ReHo changings in TOF group.

	Voxel	MNI coordinates			*T*-value
		X	Y	Z	
Right brainstem	25	12	−27	−39	4.3840
Right posterior lobe of cerebellum	23	18	−84	−39	−3.4583
Right inferior temporal gyrus	28	51	12	−42	−4.1134
Right middle occipital gyrus	26	36	−96	6	4.9977
Right inferior parietal gyrus	52	48	−45	42	5.6256
Right precuneus	24	12	−54	69	5.5039
Left precuneus	37	−15	−48	75	3.9901

*Adjusted by AlphaSim, Cluster size = 23, P < 0.01.*

*ReHo, regional homogeneity; TOF, Tetralogy of Fallot; MNI, Montreal Neurological Institute.*

**TABLE 3:** Cerebral ALFF changings in TOF group.

	Voxel	BA	MNI coordinates			*T*-value
			*X*	*Y*	*Z*	
Left cerebellum	132	18	−30	−66	−21	−4.8936
Left middle inferior occipital gyrus	122	19	−51	−75	6	−5.2099
Left inferior occipital gyrus	132	18	−21	−93	6	−4.6053
Right cerebellum	87	18	6	−81	−30	−3.6358
Left medial prefrontal cortex	88	46	−39	30	30	4.6931
Left cingulum	106	23	3	−63	21	4.7138
Right parahippocampal gyrus	94	36	30	−30	−18	4.6963

*Adjusted by AlphaSim, voxel > 65, P < 0.05.*

*ALFF, amplitude of low frequency fluctuations; TOF, Tetralogy of Fallot; MNI, Montreal Neurological Institute; BA, Brodmann area.*

After analyzing the Pearson correlations between the demographic variables and ReHo or ALFF changes in TOF children, age at surgery, postoperative time, preoperative saturation of pulse oxygen (SpO_2_), preoperative systolic blood pressure (SBP), cardiopulmonary bypass (CBP) time, and aortic occlusion (AO) time were found to be related to ReHo changes (Supplementary Table 1), and preoperative SpO_2_, CBP time, and AO time were related to ALFF changes (Supplementary Table 2).

The correlations between VIQ, FSIQ, and ReHo or ALFF changes were further determined by multiple linear regression (Table 4 and Supplementary Table 3). The results showed that only ALFF changes in the MIOG.L were positively associated with VIQ (beta = 0.908, *P* = 0.012) after adjusting for all covariates (model 3). Additionally, Table 5 shows that ReHo changes in the right precuneus (PCUN.R) and left precuneus (PCUN.L) were positively associated with ALFF changes in the MIOG.L.

**TABLE 4:** Multivariable association of cerebral amplitude of low frequency fluctuations changings and cognitive abilities in TOF postoperative children.

	VIQ		FSIQ	
	Beta (95%CI)	*p*-value	Beta (95%CI)	*p*-value
Cb. L				
Model 1	−0.644 (−199.094, 30.801)	0.119	−0.482 (−262.708, 92.762)	0.274
Model 2	−0.644 (−199.094, 30.801)	0.119	−0.482 (−262.708, 92.762)	0.274
Model 3	−0.559 (−210.423, 72.949)	0.249	−0.453 (−319.912, 148.396)	0.367
MIOG. L				
Model 1	**0.847 (38.248, 236.593)**	**0.016**	**0.800 (24.405, 326.257)**	**0.031**
Model 2	**0.847 (38.248, 236.593)**	**0.016**	**0.800 (24.405, 326.257)**	**0.031**
Model 3	**0.908 (46.240, 210.637)**	**0.012**	0.796 (−9.657, 355.872)	0.058
IOG. L				
Model 1	−0.253 (−86.195, 54.243)	0.584	−0.133 (−108.428, 85.799)	0.777
Model 2	−0.253 (−86.195, 54.243)	0.584	−0.133 (−108.428, 85.799)	0.777
Model 3	−0.213 (−86.362, 62.957)	0.686	−0.110 (−126.076, 107.490)	0.836
Cb. R				
Model 1	0.062 (−32.278, 35.964)	0.895	0.248 (−34.730, 54.677)	0.591
Model 2	0.062 (−32.278, 35.964)	0.895	0.248 (−34.730, 54.677)	0.591
Model 3	0.261 (−29.052, 43.112)	0.617	0.324 (−40.944, 67.800)	0.531
MPFC. L				
Model 1	0.449 (−33.386, 85.252)	0.312	0.474 (−42.001, 115.858)	0.283
Model 2	0.449 (−33.386, 85.252)	0.312	0.474 (−42.001, 115.858)	0.283
Model 3	0.350 (−50.086, 86.926)	0.497	0.445 (−64.586, 136.754)	0.376
Cg. L				
Model 1	0.638 (−4.989, 30.853)	0.123	0.505 (−13.307, 40.942)	0.247
Model 2	0.638 (−4.989, 30.853)	0.123	0.505 (−13.307, 40.942)	0.247
Model 3	0.521 (−13.304, 34.144)	0.290	0.485 (−22.459, 52.292)	0.330
PHG. R				
Model 1	−0.387 (−119.600, 55.682)	0.392	−0.489 (−166.466, 57.361)	0.266
Model 2	−0.387 (−119.600, 55.682)	0.392	−0.489 (−166.466, 57.361)	0.266
Model 3	−0.295 (−120.564, 76.721)	0.571	−0.464 (−193.660, 87.659)	0.355

*Model 1 adjusted for age, hospital stays, age of surgery and postoperative time.*

*Model 2 adjusted for model 1 plus CPB time and AO time.*

*Model 3 adjusted for model 2 plus preoperative SpO2, preoperative SBP, preoperative DBP and preoperative pH.*

*Cb. L, left cerebellum; MIOG. L, left middle inferior occipital gyrus; IOG. L, left inferior occipital gyrus; Cb. R, right cerebellum; MPFC. L, left medial prefrontal cortex; CG.*

*L, left cingulum; PHG. R, right parahippocampal gyrus; VIQ, verbal intelligence quotient; FSIQ, full scale intelligence quotient.*

*Bold values represent that the results have statistical significance.*

**TABLE 5:** Pearson correlation between cerebral ReHo changings and cerebral ALFF changings in TOF group.

	Cb. L	MIOG. L	IOG. L	Cb. R	MPFC. L	Cg. L	PHG. R
BS. R	0.331	−0.144	0.666	0.170	−0.595	0.058	0.377
PLC. R	−0.114	0.272	−0.248	0.577	0.346	−0.062	0.381
ITG. R	−0.628	0.280	−0.586	−0.089	0.758*	0.166	−0.080
MOG. R	0.680*	−0.492	0.679*	0.000	−0.656	0.254	−0.090
IPG. R	−0.274	0.031	−0.817**	−0.343	0.283	0.226	−0.125
PCUN. R	−0.807**	0.750*	−0.348	0.036	0.280	0.101	0.174
PCUN. L	−0.633	0.846**	−0.468	0.330	0.303	−0.235	0.218

*Correlation is significant at the 0.05 level, **Correlation is significant at the 0.01 level.

*ReHo, regional homogeneity; ALFF, amplitude of low frequency fluctuations; TOF, Tetralogy of Fallot; Cb. L, left cerebellum; MIOG. L, left middle inferior occipital gyrus; IOG. L, left inferior occipital gyrus; Cb. R, right cerebellum; MPFC. L, left medial prefrontal cortex; CG. L, left cingulum; PHG. R, right parahippocampal gyrus; BS. R, right brainstem; PLC. R, right posterior lobe of cerebellum; ITG. R, right inferior temporal gyrus; MOG. R, right middle occipital gyrus; IPG. R, right inferior parietal gyrus; PCUN.*

*R, right precuneus; PCUN. L, left precuneus; VIQ, verbal intelligence quotient.*

*Bold values represent that the results have statistical significance.*

## Discussion

Our cross-sectional study was the first to identify positive correlations between decreased ALFF values in the MIOG.L and a lower VIQ in postoperative TOF children at school age. In addition, abnormal ReHo values in the PCUN.L and PCUN.R were also positively related to ALFF values in the MIOG.L, which may compensate for the VIQ caused by low ALFF values in MIOG.L.

ALFF and ReHo, two BOLD signals, can indirectly reflect brain function (Golestani et al., 2017). Our previous study focused on the effects of structural alteration on the cognitive abilities of TOF children, indicating positive correlations between decreased cortical thickness and a low VIQ (Ma et al., 2020). Considering decreased oxygen consumption and oxygen delivery in the brain before surgery (Sun et al., 2015), close attention to neuronal activity changes should be paid in children with TOF. However, few studies have investigated the relationship between neuronal activity and cognition in TOF children. Herein, based on units of neurons, the association between neuronal activity and cognition was determined in this study, and combined with the existing research, the underlying mechanisms of a low VIQ induced by abnormal ReHo and ALFF values were also explored.

The results revealed that TOF children with lower ALFF values in the MIOG.L had a lower VIQ, indicating that the MIOG.L plays a critical role in language development. Generally, the MIOG.L is recognized as a visual processing region; however, it is also important to speech perception, including semantic and syntactic processing, language processing strategy, semantic representation, object identification, and generalization (Pigdon et al., 2019; Stroh et al., 2019; Victoria et al., 2019; Fairhall, 2020; Liu et al., 2020; Maffei et al., 2020). Different from the traditional understanding of visual regions, some studies have shown that visual processing also promotes early language development (Teinonen et al., 2008). Recently, left occipital regions were demonstrated as a visual input to language areas, which might manifest as pure alexia (without agraphia) when affected by ischemia (Sheetal et al., 2019). Additionally, a study on children with developmental language disorders showed that increased left inferior occipital volume may compensate for language regions (Pigdon et al., 2019). In addition, occipital regions can couple with other brain regions participating in many forms of verbal processing. When syntactic processing occurs, the left middle occipital regions along with the right supramarginal gyrus (SMG) are activated (Stroh et al., 2019). Moreover, population-based studies have shown that occipitotemporal (OT) regions play a crucial role in transforming visual symbols into meanings and sounds (Taylor et al., 2019; Zhou et al., 2019) and that semantic information strengthens the connection between OT regions and the left ventral inferior frontal gyrus (Wang J. et al., 2019).

Our results also showed that decreased ALFF values in the MIOG.L are positively correlated with increased ReHo values in the PCUN.L and PCUN.R, suggesting that elevated neuronal connections in the PCUN.L and PCUN.R may compensate for the low VIQ induced by inactive spontaneous activity in the MIOG.L. The PCUN, an associative region, is a part of the posteromedial parietal cortex, which engages in visuospatial imagery, episodic memory retrieval, self-processing, and consciousness by associating with many brain regions, including the thalamus, posterior cingulate, supplementary motor area, and dorsal premotor area (Cavanna and Trimble, 2006; Cunningham et al., 2017; Wang Z. et al., 2019). In addition, the parietal cortex, along with the temporal cortex and occipital cortex, connects to the temporoparietooccipital cortex (TPO), which is a highly associative cortical network and is involved in the integration of somatosensory, auditory, and visual information (Leichnetz, 2001). Additionally, many studies have reported that the PCUN is related to semantic processing and phonologic processing (Kjaer et al., 2001; Coslett and Schwartz, 2018). Similarly, our previous study suggested that the PCUN may connect with the temporal lobe *via* the temporoparietal junction (TPJ) and influence VIQ in TOF children (Ma et al., 2020). Thus, based on our results and those of previous studies, we speculate that increased neuronal connections in the PCUN.L and PCUN.R play a compensatory role in low VIQ induced by decreased ALFFs in the MIOG.L.

However, some limitations still exist in our study. First, our research should have recruited more participants to increase the reliability and generalizability of the results. Moreover, we evaluated the cognitive ability of TOF children after surgery at school age, and the specific timing of the start and maintenance of ReHo and ALFF changes is still unclear. Those children require continuous follow up. Furthermore, though recent study on 7–11 years old children who were conducted general anesthesia under 3 has shown that the exposure to general anesthesia in early childhood was not markedly related to the reduced intelligence in later stage (Schuttler et al., 2021), anesthesia was still demonstrated to be a non-negligible factor of cognitive levels in many studies (Pang et al., 2021; Shen et al., 2021; Wu and Zhu, 2021). However, we did not analyze the effect of anesthesia on cognition because of lots of anesthesia data missed. Additionally, children who were suspected of having neurodevelopmental disorders according to their guardians were more likely to be enrolled in this study, which may result in certain biases. Finally, this study was a cross-sectional study and cannot be used to determine the causal relationship between fMRI changes and cognition.

## Conclusion

In summary, compared with HC, postoperative children with TOF had a lower VIQ at school age, which was positively related to decreased ALFF values of the MIOG.L. The results revealed that preoperative brain injury in TOF children, even at school age, have persistent negative effects on neurons, especially in the MIOG.L. The possible mechanisms may be delayed language development caused by inactive spontaneous neural activity in the MIOG.L. Fortunately, these changes might be compensated by increased neural connections in the PCUN.L and PCUN.R. Therefore, it is important to pay close attention to the language development of children with TOF. Cognitive ability evaluations, especially verbal cognition, should be included in conventional postoperative reviews and follow-ups.

## Data Availability Statement

The original contributions presented in the study are included in the article/Supplementary Material, further inquiries can be directed to the corresponding author/s.

## Ethics Statement

The studies involving human participants were reviewed and approved by the Children’s Hospital of Nanjing Medical University ethics committee. Written informed consent to participate in this study was provided by the participants’ legal guardian/next of kin.

## Author Contributions

XM and MY designed the study protocol and proofread the manuscript. YH, YL, and SM collected the information and performed the data analysis under the close supervision of PZ, YP, ZY, FC, ZX, YC, and XL. SM drafted the manuscript. YP provided the help of technology. QH took charge of the modification of language. All authors contributed to the article and approved the submitted version.

## Conflict of Interest

The authors declare that the research was conducted in the absence of any commercial or financial relationships that could be construed as a potential conflict of interest. The reviewer Y-CC declared a shared affiliation, though no other collaboration, with the authors to the handling Editor.

## Publisher’s Note

All claims expressed in this article are solely those of the authors and do not necessarily represent those of their affiliated organizations, or those of the publisher, the editors and the reviewers. Any product that may be evaluated in this article, or claim that may be made by its manufacturer, is not guaranteed or endorsed by the publisher.

## Footnotes

1
www.fil.ion.ucl.ac.uk/spm

2
http://www.restfmri.net/forum/REST_V1.8
