# Supplementary material for: The Effect of Abnormal Regional Homogeneity and Spontaneous Low-Frequency Brain Activity on Lower Cognitive Ability: A Cross-Sectional Study on Postoperative Children With Tetralogy of Fallot
Source: Front Neurosci. 2022 Feb 7;15:685372. doi: 10.3389/fnins.2021.685372 (PMC8858977; doi:10.3389/fnins.2021.685372)
Supplement: Supplementary file 1 [file Table_1.docx]

Supplementary table 1 Pearson correlation between cerebral ReHo changings and demographic variables in TOF group.

|  | Age | Age of surgery | Postoperative time | Hospital stays | Preoperative SpO_2_ | Preoperative SBP | Preoperative DBP | Preoperative pH | CPB time | AO time | VIQ | PIQ | FSIQ |
| --- | --- | --- | --- | --- | --- | --- | --- | --- | --- | --- | --- | --- | --- |
| BS. R | -0.352 | **0.765^*^** | -0.534 | 0.146 | **0.760^*^** | -0.122 | 0.386 | 0.588 | -0.560 | **-0.802^*^** | -0.350 | -0.031 | -0.202 |
| PLC. R | 0.215 | -0.227 | 0.575 | -0.697 | 0.087 | 0.427 | -0.496 | -0.415 | 0.344 | 0.368 | -0.027 | 0.301 | 0.160 |
| ITG. R | 0.036 | -0.307 | 0.351 | -0.064 | -0.465 | 0.334 | -0.264 | -0.260 | 0.473 | **0.715^*^** | 0.385 | 0.112 | 0.253 |
| MOG. R | -0.212 | 0.703 | -0.742 | 0.199 | 0.592 | -0.427 | 0.179 | 0.369 | -0.641 | **-0.806^*^** | -0.326 | 0.034 | -0.150 |
| IPG. R | 0.528 | **-0.797^*^** | **0.772^*^** | -0.482 | -0.319 | 0.302 | -0.023 | -0.258 | **0.882^**^** | 0.426 | 0.162 | 0.024 | 0.117 |
| PCUN. R | -0.202 | 0.225 | -0.246 | 0.070 | 0.572 | 0.653 | 0.662 | 0.574 | 0.196 | -0.086 | **0.717^*^** | 0.258 | 0.496 |
| PCUN. L | 0.090 | -0.233 | 0.172 | -0.349 | 0.398 | **0.950^**^** | 0.299 | -0.066 | 0.375 | 0.184 | 0.605 | 0.243 | 0.432 |

* Correlation is significant at the 0.05 level, ** Correlation is significant at the 0.01 level.

ReHo, regional homogeneity; TOF, tetralogy of Fallot; BS. R, right brainstem; PLC. R, right posterior lobe of cerebellum; ITG. R, right inferior temporal gyrus; MOG. R, right middle occipital gyrus; IPG. R, right inferior parietal gyrus; PCUN. R, right precuneus; PCUN. L, left precuneus; SpO_2_, saturation of pulse oxygen; SBP, systolic blood pressure; DBP, diastolic blood pressure; pH, potential of hydrogen; CPB, cardiopulmonary bypass; AO, aortic occlusion VIQ, verbal intelligence quotient; PIQ, performance intelligence quotient; FSIQ, full scale intelligence quotient
