# Supplementary material for: The Effect of Abnormal Regional Homogeneity and Spontaneous Low-Frequency Brain Activity on Lower Cognitive Ability: A Cross-Sectional Study on Postoperative Children With Tetralogy of Fallot
Source: Front Neurosci. 2022 Feb 7;15:685372. doi: 10.3389/fnins.2021.685372 (PMC8858977; doi:10.3389/fnins.2021.685372)
Supplement: Supplementary file 2 [file Table_2.docx]

Supplementary table 2 Pearson correlation between cerebral ALFF changings and demographic variables in TOF group.

|  | Age | Age of surgery | Postoperative time | Hospital stays | Preoperative SpO_2_ | Preoperative SBP | Preoperative DBP | Preoperative pH | CPB time | AO time | VIQ | PIQ | FSIQ |
| --- | --- | --- | --- | --- | --- | --- | --- | --- | --- | --- | --- | --- | --- |
| Cb. L | 0.280 | 0.514 | -0.265 | 0.313 | -0.084 | **-0.773^*^** | -0.535 | -0.313 | -0.526 | -0.315 | **-0.669^*^** | -0.428 | -0.597 |
| MIOG. L | -0.084 | -0.082 | -0.017 | -0.517 | 0.559 | 0.684 | 0.549 | 0.524 | 0.297 | -0.049 | **0.854^**^** | 0.544 | **0.751^*^** |
| IOG. L | -0.443 | 0.552 | -0.624 | 0.352 | 0.222 | -0.599 | 0.120 | 0.420 | **-0.868^**^** | -0.625 | -0.280 | 0.004 | -0.131 |
| Cb. R | 0.222 | 0.458 | -0.100 | -0.409 | 0.415 | -0.230 | -0.445 | -0.006 | -0.248 | -0.123 | 0.383 | 0.419 | 0.428 |
| MPFC. L | -0.258 | -0.581 | 0.311 | -0.396 | -0.311 | 0.610 | -0.231 | -0.400 | 0.411 | **0.854^**^** | 0.410 | 0.522 | 0.507 |
| Cg. L | -0.266 | 0.028 | -0.231 | -0.105 | 0.436 | **0.811^*^** | 0.033 | -0.304 | 0.263 | 0.290 | -0.085 | 0.202 | 0.044 |
| PHG. R | 0.575 | 0.414 | 0.277 | -0.016 | 0.265 | -0.247 | -0.076 | 0.191 | 0.128 | -0.355 | -0.118 | -0.399 | -0.297 |

* Correlation is significant at the 0.05 level, ** Correlation is significant at the 0.01 level.

ALFF, amplitude of low frequency fluctuations; TOF, tetralogy of Fallot; Cb. L, left cerebellum; MIOG. L, left middle inferior occipital gyrus; IOG. L, left inferior occipital gyrus; Cb. R, right cerebellum; MPFC. L, left medial prefrontal cortex; CG. L, left cingulum; PHG. R, right parahippocampal gyrus; SpO_2_, saturation of pulse oxygen; SBP, systolic blood pressure; DBP, diastolic blood pressure; pH, potential of hydrogen; CPB, cardiopulmonary bypass; AO, aortic occlusion; VIQ, verbal intelligence quotient; PIQ, performance intelligence quotient; FSIQ, full scale intelligence quotient.
