# Supplementary material for: The Effect of Abnormal Regional Homogeneity and Spontaneous Low-Frequency Brain Activity on Lower Cognitive Ability: A Cross-Sectional Study on Postoperative Children With Tetralogy of Fallot
Source: Front Neurosci. 2022 Feb 7;15:685372. doi: 10.3389/fnins.2021.685372 (PMC8858977; doi:10.3389/fnins.2021.685372)
Supplement: Supplementary file 3 [file Table_3.docx]

Supplementary Table 3 Multivariable association of cerebral regional homogeneity changings and cognitive abilities in TOF postoperative children

|  | VIQ | |  | FSIQ | |
| --- | --- | --- | --- | --- | --- |
|  | Beta (95%CI) | p-value |  | Beta (95%CI) | p-value |
| BS. R |  |  |  |  |  |
| Model 1 | -0.216 (-159.912,108.260) | 0.642 |  | -0.195 (-213.302,150.358) | 0.675 |
| Model 2 | -0.216 (-159.912,108.260) | 0.642 |  | -0.195 (-213.302,150.358) | 0.675 |
| Model 3 | -0.083 (-158.089,140.253) | 0.876 |  | -0.151 (-252.508,202.596) | 0.776 |
| PLC. R |  |  |  |  |  |
| Model 1 | -0.173 (-162.302,119.237) | 0.710 |  | 0.047 (-184.876,200.597) | 0.921 |
| Model 2 | -0.173 (-162.302,119.237) | 0.710 |  | 0.047 (-184.876,200.597) | 0.921 |
| Model 3 | 0.190 (-157.616,208.581) | 0.719 |  | 0.200 (-239.633,322.297) | 0.704 |
| ITG. R |  |  |  |  |  |
| Model 1 | 0.385 (-105.088,224.571) | 0.394 |  | 0.172 (-201.426,273.509) | 0.712 |
| Model 2 | 0.385 (-105.088,224.571) | 0.394 |  | 0.172 (-201.426,273.509) | 0.712 |
| Model 3 | 0.085 (-229.290,259.316) | 0.873 |  | 0.070 (-356.978,395.227) | 0.895 |
| MOG. R |  |  |  |  |  |
| Model 1 | 0.067 (-138.167,155.303) | 0.887 |  | 0.160 (-168.418,223.555) | 0.732 |
| Model 2 | 0.067 (-138.167,155.303) | 0.887 |  | 0.160 (-168.418,223.555) | 0.732 |
| Model 3 | 0.173 (-133.613,172.179) | 0.744 |  | 0.194 (-200.721,267.557) | 0.712 |
| IPG. R |  |  |  |  |  |
| Model 1 | 0.297 (-120.809,210.248) | 0.518 |  | 0.299 (-162.399,284.150) | 0.515 |
| Model 2 | 0.297 (-120.809,210.248) | 0.518 |  | 0.299 (-162.399,284.150) | 0.515 |
| Model 3 | 0.433 (-107.702,221.455) | 0.392 |  | 0.335 (-196.861,332.174) | 0.517 |
| PCUN. R |  |  |  |  |  |
| Model 1 | 0.663 (-44.163,341.697) | 0.104 |  | 0.331 (-228.053,428.706) | 0.468 |
| Model 2 | 0.663 (-44.163,341.697) | 0.104 |  | 0.331 (-228.053,428.706) | 0.468 |
| Model 3 | 0.502 (-191.015,465.254) | 0.310 |  | 0.290 (-436.354,680.155) | 0.577 |
| PCUN. L |  |  |  |  |  |
| Model 1 | 0.156 (-105.488,139.074) | 0.738 |  | 0.137 (-145.592,185.484) | 0.769 |
| Model 2 | 0.156 (-105.488,139.074) | 0.738 |  | 0.137 (-145.592,185.484) | 0.769 |
| Model 3 | 0.259 (-101.349,149.998) | 0.620 |  | 0.165 (-173.513,221.191) | 0.754 |

Model 1 adjusted for age, age of surgery, postoperative time and hospital stays

Model 2 adjusted for model 1 plus CPB time and AO time

Model 3 adjusted for model 2 plus preoperative SpO2, preoperative SBP, preoperative DBP and preoperative pH

BS. R, right brainstem; PLC. R, right posterior lobe of cerebellum; ITG. R, right inferior temporal gyrus; MOG. R, right middle occipital gyrus; IPG. R, right inferior parietal gyrus; PCUN. R, right precuneus; PCUN. L, left precuneus; VIQ, verbal intelligence quotient; FSIQ, full scale intelligence quotient.
